# Links between COVID-19 and Parkinson’s disease/Alzheimer’s disease: reciprocal impacts, medical care strategies and underlying mechanisms

**DOI:** 10.1186/s40035-023-00337-1

**Published:** 2023-01-30

**Authors:** Pei Huang, Lin-Yuan Zhang, Yu-Yan Tan, Sheng-Di Chen

**Affiliations:** 1 Department of Neurology and Institute of Neurology, Ruijin Hospital, Shanghai Jiao Tong University School of Medicine, Shanghai, 200025 China; 2 Department of Neurology, Shanghai General Hospital, Shanghai, 200080 China; 3 Lab for Translational Research of Neurodegenerative Diseases, Shanghai Institute for Advanced Immunochemical Studies (SIAIS), Shanghai Tech University, Shanghai, 201210 China

**Keywords:** COVID-19, SARS-CoV-2, Parkinson’s disease, Alzheimer’s disease, Mechanism

## Abstract

The impact of coronavirus disease 2019 (COVID-19) pandemic on patients with neurodegenerative diseases and the specific neurological manifestations of COVID-19 have aroused great interest. However, there are still many issues of concern to be clarified. Therefore, we review the current literature on the complex relationship between COVID-19 and neurodegenerative diseases with an emphasis on Parkinson’s disease (PD) and Alzheimer’s disease (AD). We summarize the impact of COVID-19 infection on symptom severity, disease progression, and mortality rate of PD and AD, and discuss whether COVID-19 infection could trigger PD and AD. In addition, the susceptibility to and the prognosis of COVID-19 in PD patients and AD patients are also included. In order to achieve better management of PD and AD patients, modifications of care strategies, specific drug therapies, and vaccines during the pandemic are also listed. At last, mechanisms underlying the link of COVID-19 with PD and AD are reviewed.

## Background

The coronavirus disease 2019 (COVID-19) pandemic induced by the severe acute respiratory syndrome coronavirus 2 (SARS-CoV-2) poses a great threat to the lives of humans all over the world. SARS-CoV-2 is an RNA virus that infects the respiratory system in humans and causes serious illness, including severe pneumonia and neurological disorders [1, 2]. Accumulating evidence suggests that COVID-19 infection might interfere with Parkinson’s disease (PD) and Alzheimer’s disease (AD), leading to worsening of symptoms or even acceleration of disease progression [3–7]. Coronaviruses can be detected in the central nervous system (CNS) of patients with PD and AD [8]. COVID-19 infection develops as the glycoprotein spike of the virus binds to angiotensin-converting enzyme 2 (ACE2) receptors, which are widespread in the brain. COVID-19 could lead to accelerated aging in the brain [9, 10]. Furthermore, patients with PD and AD have been reported with higher risks of COVID-19 infection, hospitalization, and mortality, indicating a close link between COVID-19 and neurodegenerative diseases [11–14].

Recently, the impact of the COVID-19 pandemic on patients with neurodegenerative diseases, as well as the specific neurological manifestations of COVID-19, has aroused great interest [15, 16]. However, there are still many issues of concern to be clarified. For example, it remains unclear how COVID-19 infection affects the symptom severity, disease progression, and neurodegeneration-related mortality, and whether COVID-19 infection could trigger neurodegenerative diseases. And vice versa, it remains uncertain how neurodegenerative diseases impact COVID-19, susceptibility to SARS-CoV-2 infection and prognosis of COVID-19 in patients with neurodegenerative diseases. Furthermore, the COVID-19 pandemic has profoundly changed the medical care model for PD and AD patients. Drug therapies for neurodegenerative diseases were also reported to impact the COVID-19 infection, and vaccines might interfere with neurodegenerative diseases. Thus, comprehensive modification of care strategies in patients with neurodegenerative diseases during the pandemic and a summary of drug therapies and vaccines related to the neurodegenerative diseases are very necessary for the management of these patients. In addition, mechanisms underlying the link between COVID-19 and neurodegenerative diseases need to be revealed. Therefore, we sought to review the current literature on the complex relationship between COVID-19 and neurodegenerative diseases with an emphasis on PD and AD.

## COVID-19 and PD

The associations between COVID-19 and PD are listed below, with highlights of the link summarized in Table 1.

**Table 1:** Highlights of the link between COVID-19 and Parkinson’s disease

Subject	Topic	Highlight
Impact of COVID-19 on PD	Effect on motor and non-motor symptoms	1. Worsening of motor symptoms, such as rigidity and tremor 2. Experiencing motor fluctuations 3. Triggering new motor symptoms 4. Worsening non-motor symptoms, such as mood, sleep, cognition, dysautonomia and hallucination 5. COVID-19 has harmful effects on motor and non-motor symptoms in PD patients both directly and indirectly
	Effect on disease progression	1. Only one retrospective longitudinal study revealed increased motor symptom progression of PD during the COVID-19 pandemic
	Effect on PD-related mortality	1. Increased PD-related mortality rates during the COVID-19 pandemic 2. PD with older age, advanced course, reduction in medication and comorbidities are more likely to have increased risk of mortality 3. Negative findings indicated that the mortality of COVID-19 in PD patients does not differ from the general population
	Trigger for PD	1. Cases have been reported with development of parkinsonism after COVID-19 infection 2. Infection of dopaminergic neurons with the H1N1 influenza virus results in aggregation of α-synuclein 3. Viral infections trigger α-synucleinopathies in animal models 4. COVID-19 infection could trigger neurodegeneration with mechanisms not clearly determined
Impact of PD on COVID-19	Vulnerability to COVID-19 infection	1. Possibly higher risks of COVID-19 infection and hospitalization 2. Contrary results were also reported that PD do not differ from general population in the COVID-19 risk
	Prognosis of COVID-19 infection	1. Enhanced risk of disease severity and mortality in PD patients than in non-PD patients 2. Inconsistent results were also reported that the prognosis of COVID‐19 patients seems comparable in patients with PD and those without it
Management of PD during COVID-19 pandemic	Modification of care strategies in PD	1. Pandemic profoundly changes the way of PD management 2. Telemedicine services with digital-visits, e-rehabilitation, and remote programming are accessible and efficient for PD during the pandemic
	Potential impact of drug therapies for PD on COVID-19	1. Amantadine can be potentially used for the prevention of COVID-19 2. Levodopa has potential impacts on SARS-CoV-2 3. Dopamine agonists may worsen the outcomes of patients with COVID-19 infection 4. Entacapone may be a potential drug against SARS-CoV-2 5. Clozapine treatment is linked with an increased risk of COVID-19 infection 6. Vitamin D supplementation is identified as a protective factor for COVID-19 infection
	Effect of COVID-19 vaccines on PD	1. COVID-19 vaccines were known or expected not to interact with PD 2. Types or incidence of side effects of vaccines in PD seem no different from the general population 3. Case reports of developing severe dyskinesia or worsening of parkinsonian symptoms after receiving vaccines 4. A case report of improvement of motor and non-motor symptoms after receiving vaccines 5. Amantadine was hypothesized of potential interference with COVID-19 vaccines
Mechanisms of the link between COVID-19 and PD		1. SARS-CoV-2 virus enters the CNS through olfactory bulb, by axonal transport from peripheral nerves, or by the hematogenous pathway via BBB 2. Systemic inflammation and sepsis which promote hypercoagulable response to form clots in brain vessels, and cytokine storm leading to hyper-inflammation and neuroinflammation 3. SARS-CoV-2 could bind to ACE2 receptors on dopaminergic neurons, which might cause neuroinflammation, excessive oxidative stress, abnormal immune response and pathological α-synuclein accumulation, leading to dopaminergic neuronal death

*ACE2* Angiotensin-converting enzyme 2; *BBB* Blood brain barrier; *CNS* Central nervous system; *COVID-19* Coronavirus disease 2019; *PD* Parkinson’s disease; *SARS-CoV-2* Severe acute respiratory syndrome coronavirus 2

### Impact of COVID-19 on PD

#### Effect on motor and non-motor symptoms

The COVID-19 pandemic is considered to worsen the motor and non-motor symptoms of PD patients, and trigger new symptoms or problems by direct and indirect ways.

Infection is a common cause for the exacerbation of parkinsonian symptoms [17]. A severe infection such as COVID-19 could have a direct harmful impact on PD motor symptoms, which deteriorate during the period of systemic inflammation [18]. A large online study (Fox Insight) has investigated the effect of the COVID-19 pandemic on PD patients [3]. Data were collected from 5429 PD patients with 51 reporting COVID-19 positive diagnoses in the US. Among those infected, 55% reported worsening of at least one existing motor symptom and 18% reported occurrence of new motor symptoms [3]. New occurrence or worsening of non-motor symptoms was also noted, including mood symptoms (71%), sleep disruptions (62%), cognitive problems (49%), and dysautonomia (38%) [3]. However, the underlying mechanism of the direct harmful effect of COVID-19 on PD is still unclear. Possible explanations include insufficient response to dopaminergic drugs after infection, altered transport of dopaminergic drugs through the blood–brain barrier (BBB) after systemic infection, and altered dopamine metabolism and receptor signaling due to the response to proinflammatory cytokines. Inflammatory processes outside the brain may lead to the boosting of pre-existing neuroinflammatory processes in PD [19].

COVID-19 also affects motor and non-motor symptoms in PD through indirect ways, such as dramatic change in routine life, social isolation, stress, anxiety and prolonged immobility [20]. In the Fox Insight online study, PD patients without COVID-19 reported disrupted medical care (64%), exercise (21%), and social activities (57%), and worsening of motor (43%) and non-motor (52%) symptoms during the pandemic [3]. An online survey of 498 PD patients in the Netherlands showed that 46.6% of PD patients were less physically active since the COVID-19 pandemic, leading to the worsening of PD symptoms (including rigidity, fatigue, tremor, and pain). Patients with a higher level of COVID-19-related stress experience severer PD symptoms, and this effect is mediated by the degree of psychological distress [21]. A survey of 733 COVID-19-negative PD patients in Tuscany reported worsening of motor symptoms (29.6%), mood problems (24.7%), and insomnia (22.2%) during the COVID-19 pandemic [22]. In a telephone-based survey of 568 Spanish patients, 65.7% of patients reported worsening of their symptoms (bradykinesia 47.7%, sleep problems 41.4%, rigidity 40.7%, gait disturbances 34.5%, anxiety 31.3%, pain 28.5%, fatigue 28.3%, depression 27.6%, tremor 20.8%, and appetite disorders 13.2%) during the pandemic [23]. We also conducted an online survey of 1764 PD patients in China during the COVID-19 pandemic in 2022 (unpublished data). In this survey, 200 PD patients had experienced isolation and only 3 of them reported COVID-19 positive diagnoses. The results showed that 50% of the PD patients reported worsening of symptoms during the pandemic, and this percentage increased to 61% among isolated PD patients. For details, severer motor symptoms during the pandemic were reported by the PD patients, including tremor (21.7%), bradykinesia (37.9%), rigidity (30.5%), and postural disability (20.1%), especially in isolated PD patients (26.5%, 46.5%, 35.5%, and 24.5%, respectively). The top three aggravated non-motor symptoms in PD patients during the pandemic included constipation (27.4%), fatigue (23.8%), and sleep disturbance (23.1%), and these proportions increased among the isolated patients (31.5%, 27.5%, and 32.5%, respectively). The data suggested that a majority of PD patients experience the deterioration of symptoms during the COVID-19 pandemic without infection. This phenomenon may be explained by a marked reduction in physical activities, increased psychological stress, and difficulties in getting access to dopaminergic drugs or medical care due to the COVID-19 lockdown [20].

In conclusion, COVID-19 exacerbates motor and non-motor symptoms in PD patients both directly and indirectly. PD patients experiencing activity restriction during the pandemic have increased rates of symptom deterioration than usual, regardless of COVID-19 positivity. The indirect effects of COVID-19 might be more detrimental than the virus itself since infection only occurs in a small number of PD patients but the COVID-19 pandemic could indirectly affect a larger population.

#### Effect on disease progression of PD

Most of the present literature on COVID-19 and PD focuses on the impact of infection itself and its effects on motor and non-motor symptoms in PD. However, whether COVID-19 has an influence on the disease progression of PD remains unclear. Only one retrospective longitudinal study focused on the disease progression rate of PD during the COVID-19 pandemic. In that retrospective longitudinal study of 264 PD patients in Switzerland, motor disease progression measured by the third part of the Unified Parkinson Disease Rating Scale (UPDRS-III) of the International Parkinson and Movement Disorders Society (MDS) was compared before versus during the COVID-19 pandemic [4]. Significant worsening of motor symptoms and increased motor disease progression were observed during pandemic-related restrictions as compared to before the COVID-19 pandemic [4]. Additionally, a trend analysis of the yearly evolution of motor symptoms in 755 PD patients from 2016 to 2021 showed that in contrast to the slow progression of mean MDS-UPDRS III scores from 2016 till the onset of the pandemic crisis (beginning of 2020), there was a steep, pharmacotherapy-independent increase from 2020 onwards (during the crisis) while the mean levodopa equivalent dose remained unchanged between 2016 and 2021[4]. This study demonstrated that the increase of symptom progression is not due to a potential reduction in medication. The COVID-19 pandemic, during which PD patients may go through immobility, social isolation, and psychological stress, may exacerbate motor symptoms and increase disease progression [4].

#### Effect on PD-related mortality

A study in Italy investigated the long-term trends and impact of COVID-19 pandemic waves on PD-related mortality [11]. In that study, 13,746 PD-related deaths (2.3% of all deaths) were identified during 2008–2020, with proportional mortality increased from 1.9% (2008) to 2.8% (2020) [11]. The PD-related mortality rate when considering PD as one among multiple causes reported in death certificates during 2020 was 28%, showing two peaks corresponding to the first (March to May, 28%) and second (October to December, 59%) pandemic waves that occurred in Italy [11]. Generally, PD-related mortality rates were steeply increased during the COVID-19 pandemic [11]. A retrospective review of 70 PD inpatients in New York found that PD patients with COVID-19 infection had a higher mortality rate compared to those not infected (35.8% vs 5.9%, *P* = 0.028) [24]. Age older than 70 years, advanced stage of PD disease, and reductions of medication were risk factors for higher mortality rate [24]. Furthermore, a multicenter study of 117 community-dwelling PD patients with COVID-19 in Italy, Iran, Spain, and the UK reported an overall mortality of 19.7%, with dementia, hypertension, and advanced PD increasing the risk of mortality [25]. In all, PD-related mortality rates have increased significantly during the COVID-19 pandemic. Patients with older age, advanced course, reduction of medication, and comorbidities are more likely to have increased risk of mortality.

However, negative findings have also been reported. In a case–control survey of 1486 PD patients and 1207 family members (controls) from Italy, 6 patients (5.7%) and 7 family members (7.6%) died from COVID-19. Mortality and COVID-19 risk in this cohort of PD patients did not differ from the general population [26]. The mortality rate was probably under-represented as this study which only enrolled community-dwelling PD patients, while patients living in nursing homes or other long-term care facilities where outbreaks with high mortality rates had been reported were excluded.

#### Trigger for PD

A meta-analysis of the impact of viral and bacterial infections on the risk of developing PD indicated that individuals with infection had a 20% increased risk of PD compared with controls [27]. Méndez-Guerrero and colleagues [28] were the first to report a 58-year-old patient who developed an asymmetric hypokinetic-rigid syndrome with hyposmia and ocular abnormalities after SARS-CoV-2 infection. Dopamine transporter single-photon emission computed tomography demonstrated an asymmetric decrease of presynaptic dopamine uptake within the putamen. Parkinsonian symptoms were improved spontaneously without any specific treatment [28]. Furthermore, Cohen and colleagues [29] reported a 45-year-old patient infected with SARS-CoV-2 who developed moderate rigidity, bradykinesia, tremor, slightly slow gait, and hypophonia. ^18^F-Fluorodopa (^18^F-FDOPA) positron emission tomography (PET) scan showed asymmetrically decreased ^18^F-FDOPA uptake in both putamens. The motor symptoms were improved after treatment with pramipexole and biperiden [29].

SARS-CoV-2 infection is considered responsible for the development of parkinsonism. However, the mechanism by which COVID-19 triggers neurodegeneration remains to be determined. Some evidence has suggested the causal link between SARS-CoV-2 infection and parkinsonism [30, 31]. Infection of dopaminergic neurons with the H1N1 influenza virus results in the aggregation of α-synuclein, which is the major protein component of Lewy bodies in the brain [32]. In animal models, viral infections can trigger α-synucleinopathies [33]. It has been reported that SARS-CoV-2 is able to enter the brain and trigger the release of inflammatory mediators [34] that are known to play a role in neurodegeneration. SARS-CoV-2 can enter the brain by invading the olfactory bulb, by axonal transport from peripheral nerves, and by hematogenous pathways through the BBB [35]. SARS-CoV-2 could bind to ACE2 receptors on dopaminergic neurons, altering the rate of accumulation of misfolded α-synuclein, and promoting mitochondria stress, autophagy, and apoptosis [35, 36]. Apart from the direct invasion of SARS-CoV-2 into the CNS, post-infection immune-mediated process also plays an important role in the development of PD [37]. Systemic effects such as vascular insults in the nigrostriatal system could lead to subsequent parkinsonism [38]. Furthermore, the cytokine storm associated with severe COVID-19 infection could trigger neuroinflammation and result in neurodegeneration [39].

### The impact of PD on COVID-19

#### Susceptibility to COVID-19 infection

A meta-analysis has found that patients with neurological disorders have a doubled risk of COVID-19 and a 40% higher risk of hospitalization for COVID-19 [40]. Furthermore, a cohort study has focused on the risk of SARS-CoV-2 infection, and hospitalization for COVID-19 in PD patients during a 15-month period of the COVID-19 pandemic [12]. The study revealed a higher risk of SARS-CoV-2 infection in PD patients (hazard ratio [HR], 1.3; 95% confidence interval [CI], 1.04–1.7) compared to matched controls. Adjusted HR of hospitalization for COVID-19 was 1.1 (95% CI 0.8–1.7) in PD [12]. Antonini et al. analyzed the outcomes of 10 PD patients infected with COVID-19 and concluded that PD patients of older age with longer disease duration are particularly susceptible to COVID-19 with a substantially high mortality rate (40%) [41]. Studies have suggested that PD patients may have an increased risk of COVID-19, mainly due to the fact that PD mostly affects elderly people with numerous comorbidities and multidrug therapies [42]. Moreover, respiratory muscle rigidity, impairment of cough reflex, and dyspnea are very common during the course of PD, possibly causing a more severe infection of COVID-19 and an increased risk of hospitalization in PD [41, 42].

Nevertheless, there are also some opposite results. Jon Stoessl et al. mentioned in a recent editorial that there was no evidence that patients with movement disorders were at increased risk of COVID-19 infection, compared to individuals with similar age and comorbidities [43]. In a single-center case–control survey of 105 PD patients and 92 controls identified as COVID-19 cases, COVID-19 risk and mortality in PD patients did not differ from the general population [26]. Fasano et al. reported that COVID-19 risk, morbidity, and mortality in patients with mild to moderate PD do not differ from the general population [26]. This discrepancy may be caused by differences in enrollment and screening criteria for participants. More studies with larger sample sizes at different stages of PD across different centers are needed to clarify whether PD increases the risk of COVID-19 infection.

#### Prognosis of COVID-19 infection

A nationwide cross-sectional study of 5,210,432 inpatients from 1468 hospitals in Germany has collected data from 64,434 PD patients, with 693 being COVID-19-positive [44]. The COVID-19-positive inpatients with PD showed higher incidence of comorbidities than non-PD COVID-19-positive subjects, particularly hypertension and chronic kidney disease [44]. In addition, the COVID-19 inpatient mortality rate was much higher in PD patients than in non-PD patients (35.4% vs 20.7%, *P* < 0.001), especially in patients aged 75–79 years [44]. In addition, a meta-analysis of 12 studies with 103,874 COVID-19 patients showed that PD is associated with an enhanced risk of disease severity (odds ratio [OR], 2.61; 95% CI 1.98–3.43) and mortality (relative risk [RR], 2.63; 95% CI 1.50–4.60) from COVID-19 [45]. Furthermore, a cohort study included 1294 Dutch residents with COVID-19 and 17,999 residents without COVID-19 identified risk factors for 30-day mortality for COVID-19 [46]. For residents with COVID-19, being male (HR, 1.8), having dementia (HR, 1.3), and having PD (HR, 1.7) are all associated with higher 30-day mortality [46]. Being male is also associated with higher mortality (HR, 1.7) in the controls, whereas having dementia and having PD are not. Therefore, having dementia and having PD are recognized as unique risk factors for mortality in COVID-19 patients [46].

However, inconsistent results have also been reported. A cohort study by Vignatelli and colleagues assessed the risk of hospitalization for COVID-19 and death in 696 PD patients compared with 8590 controls. The 3-month hospitalization rate for COVID-19 was 0.6% in PD and 0.7% in controls. The 30-day risk of death after hospitalization was high in both cohorts (around 35%) without a difference between the two groups [47]. A meta-analysis of 13 studies found that the prognosis of COVID-19 in patients with PD is comparable to that in patients without PD [48]. The prognosis for COVID-19 varies extremely and is affected by many factors, such as the COVID-19 infection ascertainment method, age, geographical context, and capacity of the healthcare system. It was hard to compare the different studies since adjustment for age and other baseline demographics, such as the history of drug administration, was not approached in the study. The prognosis of COVID-19 in PD patients should be further confirmed in larger population-based cohorts with adjustment of interfering factors.

### Management of PD during the COVID-19 pandemic

#### Modification of care strategies for PD

The COVID-19 pandemic has profoundly affected the delivery of in-person medical care for PD patients. Digital rehabilitation strategies including virtual rehabilitation platforms as an alternative to deliver rehabilitation services at the community level should be encouraged [49]. Although telemedicine services are not superior to the in-person visits in providing care, a growing body of evidence suggests that it offers greater efficiency and service for PD patients [50]. An online survey in Italy has investigated the impact of COVID-19 on access to telehealth care among 197 PD patients and 42 neurologists [51]. The results showed that 37.6% of PD patients and 88.1% of neurologists had chosen alternatives to in-person visits, while 13.7% of PD patients and 40.5% of neurologists used telemedicine. Most of them were satisfied with the use of telemedicine during the COVID-19 pandemic, indicating that telemedicine has the potential to improve the care for PD patients, especially when access to in-person visits is limited [51]. Another survey of 417 PD patients in Canada aiming to see the effects of confinement on patients’ daily living and disease management suggested that COVID-19-related confinement affected PD manifestation and management [52], and the patients reported varying levels of interest in continuing remote care via phone or video conference [52].

The online dancing program is one option for telemedical care. Morris et al. conducted an observational study in Australia to evaluate the impact of online therapeutic dancing classes in early- to mid-stage PD patients. Thirteen participants completed 8 one-hour sessions of online therapeutic dancing and were able to quickly adapt to online delivery with support and resources. This study showed that online dance therapy is safe and beneficial to early adopters during the pandemic [53]. Another online survey explored the accessibility and benefit of home-based dance programs for PD patients. Data from 276 individuals with PD showed that 94.9% of participants benefited from home-based dance programs, including improvements in physical (balance and posture) and non-physical (mood and confidence) aspects. A great portion (70.8%) of the participants expressed strong preference for continuing with home-based practice in the future. These results indicated that at-home dance is accessible and beneficial for PD patients, and digital dance programs are potential therapies for PD [54].

Moreover, for PD patients who have received deep brain stimulation (DBS) therapy, remote programming greatly facilitates on-time follow-up and timely programming, which are important for high-quality management of PD. With the use of internet technologies, Xu et al. continued to provide motor and non-motor symptom assessment and remote programming services for 36 postsurgical DBS patients during the COVID-19 pandemic [55]. The patients showed significant improvements in UPDRS-III score and most of them were willing to use remote programming again [55]. Thus, remote programming based on online evaluation of symptoms is critical for postsurgical management of DBS patients with PD during the COVID-19 pandemic.

In the COVID-19 pandemic, telemedicine that offers digital visits, e-rehabilitation and remote programming can break the obstacles of limited access to routine-visits in hospitals.

#### Potential impact of drug therapies for PD on COVID-19

Importantly, a number of anti-PD medications might have potential benefits against SARS-CoV-2. The PD medication amantadine is also approved by Food and Drug Administration (FDA) as a therapy against the influenza A virus, and its antiviral properties make it a potential treatment for COVID-19 [56]. A small number of COVID-19-positive PD patients taking amantadine did not manifest symptoms of COVID-19 [42] and another case report has demonstrated similar results [57]. A hospital-based, observational, retrospective cohort study collected data from 256 PD patients (including 87 patients taking amantadine and 169 patients without amantadine) by an online questionnaire survey [58], and found that the rate of COVID-19 disease was 5.7% in patients taking amantadine and 11.8% in patients without amantadine [58]. Amantadine is also associated with a significantly reduced risk of COVID-19 infection (OR, 0.26; 95% CI 0.07–0.89) [58]. It is hypothesized that amantadine may target SARS-CoV-2 by disrupting lysosomal gene expression [59]. Therefore, amantadine may be potentially used for the prevention of COVID-19.

Other anti-PD medications, such as levodopa, may have an impact on SARS-CoV-2 since alterations of the dopamine synthetic pathway are considered to be involved in the pathophysiology of SARS-CoV-2 [60]. Use of dopamine agonists has been found to be associated with worse outcomes of patients with COVID-19 infection [26, 61]. In addition, in an interactome analysis of potential drugs repurposed for SARS-CoV-2, entacapone was identified as a potential drug [62]. Clozapine is frequently used and recommended to manage psychosis or dyskinesia in PD. In a study of 6309 participants including 102 persons positive with COVID-19, clozapine treatment was linked with an increased risk of COVID-19 infection compared with other antipsychotics [63].

Interestingly, vitamin D supplementation was identified as a protective factor against COVID-19 infection in PD patients (OR, 0.50; 95% CI 0.30–0.83) [64]. Regular vitamin D3 consumption of 2000–5000 IU/day may reduce the risk and severity of COVID-19 in PD patients [65]. Vitamin D plays a protective role in the development and progression of PD, in addition to its potent antiviral effects [66]. PD patients usually have decreased levels of 25-hydroxy vitamin D3 compared to controls, and low concentrations of 25-hydroxy vitamin D3 are linked to higher incidence and greater severity of the COVID-19 disease [66]. Administration of vitamin D3 significantly improves the motor and non-motor manifestations of PD [67]. Vitamin D deficiency has been linked to complications in patients with SARS-CoV-2 infection and PD, while supplementation of vitamin D3 in PD patients can help minimize the risk and burden of COVID-19 complications [67]. Vitamin D may directly down-regulate the ACE2 receptor, the major receptor for viral entry, thereby lowering the probability of COVID-19 infection [68].

#### Effect of COVID-19 vaccines on PD

The approved mRNA-based vaccines and viral vector vaccines under development are known or expected not to interact with the neurodegenerative process in PD [69]. Types or incidence of side effects of these vaccines in PD showed no difference from the general population [69]. The vaccines also seem to be safe for older adults, but cautions are needed for the specific subgroup of very frail and terminally ill elderly persons with PD living in long-term care facilities [69]. Erro et al. reported 2 PD patients who developed severe dyskinesia after receiving the BNT162b2 (Pfizer/BioNTech) mRNA vaccine [70]. The reasons were not clear, but systemic inflammatory response may be a trigger. Innate immune response following mRNA vaccination is critical for the initiation of adaptive immunity. This highlights the variability of response triggered by the vaccine that depends on individual immunological profiles [70]. Imbalzano et al. [71] also observed 2 PD patients who showed worsening of parkinsonian symptoms after receiving the third vaccine dose (mRNA-1273 booster). The two patients and other cases of vaccine-related movement disorder completely recovered after a few days with parkinsonian therapy modifications, symptomatic treatment, or even spontaneously [71]. Conversely, Contaldi et al. described a 55-year-old PD patient who benefited from administration of the mRNA-1273 vaccine [72]. Right after the first shot, the patient reported global improvement in motor and non-motor symptoms and a sustained benefit for almost one week after the second shot [72]. However, the mechanisms underlying these beneficial effects are not easy to clarify. Taken together, COVID-19 vaccination with approved vaccines for persons with PD is recommended, unless there is a specific contraindication [69].

One more thing that needs caution for amantadine is the hypothesized potential interference with COVID-19 vaccines [73]. For influenza A, the mechanisms of action of amantadine are considered to be related to the interference with the endosome, thus interrupting the release of virions into the cell [56]. While current COVID-19 mRNA vaccines use lipid nanoparticles as critical components for transporting mRNA into host cells, cautions should be paid to the lipophilic properties of amantadine and its ability to interrupt the endosome, which may interfere with the release of the mRNA into the cell matrix and subsequently with its binding to ribosomes [56, 74]. As more people are going to be vaccinated and more similar vaccines are going to be introduced, for PD patients treated with amantadine, vaccination approach should be selected with caution.

### Mechanisms of the link between COVID-19 and PD

**Fig. 1:**
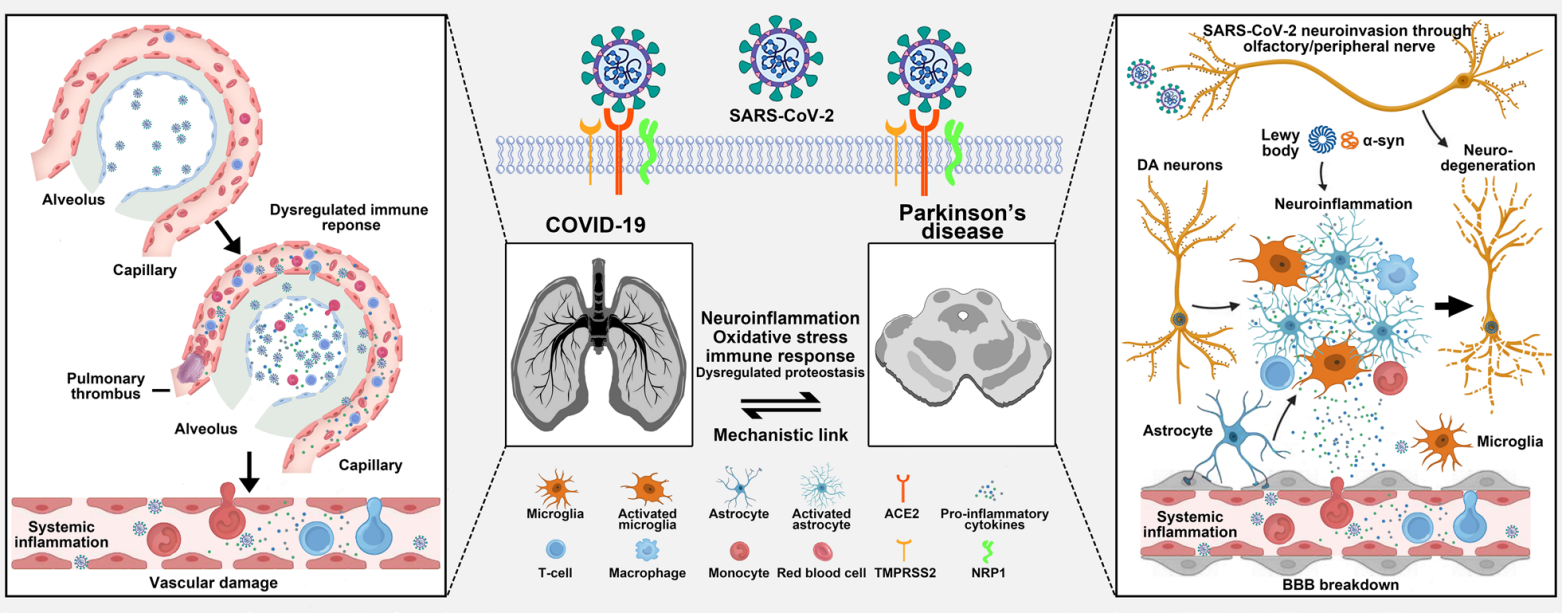
Putative mechanisms of the link between COVID-19 and Parkinson's disease (PD). With the aid of TMPRSS2, the S protein of SARS-CoV-2 binds with ACE2 to enter the host cell. The SARS-CoV-2 virus may affect the midbrain in the following ways: vascular damage (BBB breakdown), systemic inflammation, and direct neuroinvasion, which then induce neuroinflammation, excessive oxidative stress, abnormal immune response, and aggregation of α-synuclein, leading to dopaminergic neuronal death and PD. ACE2: angiotensin-converting enzyme 2; α-syn: a-synuclein; BBB: blood–brain barrier; COVID-19: Coronavirus disease 2019; NRP1: neuropilin 1; SARS-CoV-2: severe acute respiratory syndrome coronavirus 2; TMPRSS2: transmembrane serine protease 2

It is already known that SARS-CoV-2 infection occurs through the cellular surface protein ACE2 and transmembrane serine protease 2 (TMPRSS2) [75]. Dopaminergic neurons are considered to be susceptible to SARS-CoV-2 infection since both ACE2 and TMPRSS2 receptors are overexpressed in the substantia nigra [76, 77]. Under certain pathological circumstances, SARS-CoV-2 acts as a neurodegenerative enhancer to potentially drive the development or progression of PD and its related motor and non-motor symptoms [35, 78]. In direct pathways, SARS-CoV-2 enters the CNS through the olfactory nerve via axonal transport from peripheral nerves, or through the BBB [79]. COVID-19 infection may increase cytokine production, leading to the activation of microglial cells, increasing T-cell activation-related immunity, and increasing neuroinflammation [38, 80, 81]. SARS-CoV-2 could bind to ACE2 receptors on dopaminergic neurons, which might alter the rate of accumulation of misfolded α-synuclein, induce mitochondria stress, affect autophagy and promote apoptosis [82, 83]. In indirect pathways, SARS-CoV-2 could lead to neurodegeneration via systemic effects. Systemic inflammation and sepsis would promote a hypercoagulable response to form clots in brain vessels, and cytokine storms could lead to hyper-inflammation and neuroinflammation [38, 84, 85] (Fig. 1).

#### Neuroinflammation

COVID-19 infection may increase cytokine production leading to the activation of microglial cells, increasing T-cell activation-related immunity, and increasing neuroinflammation [38, 80, 81]. A neuropathological study of 43 COVID-19 patients revealed microglial activation and invasion of cytotoxic T cells in the brainstem, which are also neuropathological signs associated with PD [86]. Microglial cells are the major resident innate immune inflammatory cells in the brain and can produce proinflammatory cytokines upon activation. Activation of microglial nucleotide-binding oligomerization domain-like receptor containing pyrin domain 3 (NLRP3) inflammasome plays a critical role in dopaminergic neurodegeneration in the mouse model of PD induced by 1-methyl-4-phenyl-1,2,3,6-tetrahydropyridine [87]. Activation of the inflammasome is not exclusively a brain event. Systemic activation of the inflammasome is likely to be involved in the formation of severe cytokine storms, which are capable of disrupting the BBB, and inducing microglial activation and neuroinflammation. The nuclear factor erythroid 2-related factor 2 (NRF2) is a transcription factor that increases the expression of a number of antioxidant proteins and suppresses the NLRP3 inflammasome [88, 89]. NRF2 activation may be a potential therapeutic approach to counteracting the NLRP3 inflammasome, which can be used for PD and COVID-19 by simultaneously reducing neuroinflammation and systemic inflammation [90].

#### Oxidative stress

Oxidative stress is a crucial mechanism related to both the cause and the progression of PD [91, 92]. Oxidative stress generates reactive oxidative species (ROS) and leads to the apoptosis of dopaminergic neurons through activation of caspases and nuclear factor κB signaling [91, 92]. Oxidative stress has also been linked to other processes involved in dopaminergic neuron degeneration, including mitochondrial dysfunction, inflammation, and excitotoxicity [91, 92]. COVID-19 is responsible for severe acute respiratory insufficiency, subsequent hypoxemia, sepsis-induced hyper-coagulation, local thrombosis in brain vessels with hypoperfusion, and cerebral hypoxia. COVID-19 might exacerbate oxidative stress dysregulation at the cellular level and promote neurodegeneration [78]. Binding of SARS-CoV-2 to ACE2 receptors on microglia decreases mitochondria energy and activates nicotinamide adenine dinucleotide phosphate oxidase, which generates ROS, promoting oxidative stress and neuroinflammation, leading to apoptosis of neighboring dopaminergic neurons [93–95].

#### Immune response

Immune cell circulation to the CNS is restricted to particular immune subsets (innate and adaptive immune cell subsets), which can take charge of the immune control in the CNS [96]. Upon viral infection, the level of immune cell migration is increased. In PD patients, immune changes occur not only in the brain, involving microglia, but also in the periphery, with changes in the innate immunity such as monocytes and the adaptive immune system, particularly T-cells [97]. The pro-inflammatory CD4+ T cells secret pro-inflammatory cytokines to activate M1 microglia, resulting in sustained neuroinflammation [97, 98]. Microglia-mediated neuroinflammation, together with activated astrocytes, acts as a link between SARS-CoV-2 and PD pathogenesis [99].

#### Dysregulated proteostasis

The hallmark of the neurodegenerative process in PD is represented by pathological accumulation of alpha-synuclein (α-syn) protein, leading to formation of Lewy bodies. This protein aggregation spreads from neuron to neuron, disrupting dopaminergic transmission and function, eventually leading to neuronal death [100]. Neuroinvasion of SARS-CoV-2 could lead to elevation of α-syn level. The aggregation of α-syn is accelerated upon the presence of the SARS-CoV-2 nucleocapsid protein (N-protein) in a test tube in vitro, suggesting a potential link between SARS-CoV-2 and α-syn that influences PD pathology [101]. Meanwhile, excessive oxidative stress correlated with SARS-CoV-2 infection can also promote the pathological aggregation of α-syn, leading to dopaminergic neurotoxicity and PD [102].

## COVID-19 and AD

The associations between COVID-19 and AD are listed below. Highlights of the link between COVID-19 and AD are summarized in Table 2.

**Table 2:** Highlights of the link between COVID-19 and Alzheimer’s disease

Subject	Topic	Highlight
Impact of COVID-19 on AD	Effect on dementia severity	1. COVID-19 and prolonged hypoxia would exacerbate severity of pre-existing cognitive impairment of AD 2. COVID-19 pandemic and related restriction aggravate cognitive impairment in AD
	Effect on neuropsychiatric symptoms	1. Clinical presentations of COVID-19 in AD patients are atypical, and neuropsychiatric symptoms are common 2. COVID-19 leads to the worsening of pre-existing neuropsychiatric symptoms in AD 3. COVID-19 pandemic and related restriction aggravate neuropsychiatric symptoms in AD 4. Worsened hyperactivity and bizarre behaviors emerge in AD animal model after isolation
	Effect on disease progression	1. There is no consensus on the effect of COVID-19 pandemic and related restrictions on dementia progression in AD patients
	Trigger for AD	1. AD-like features are involved in COVID-19 neuropathology 2. SARS-CoV-2 intrudes brain structure and causes brain functional abnormalities at 6-month longitudinal follow-up 3. Increased risks of memory problems and AD are shown at 12 months following acute COVID-19 infection 4. COVID-19-related social isolation and loneliness increase the risk of cognition decline and future dementia
Impact of AD on COVID-19	Vulnerability to COVID-19 infection	1. AD patients are at increased risk of COVID-19 infection 2. Biological and socioeconomic factors work together to make individuals with AD vulnerable to COVID-19 infection
	Prognosis of COVID-19 infection	1. Pre-existing dementia is associated with the largest risk of COVID-19 hospitalization and mortality 2. Age, comorbidities, *APOE* ε4 allele, and *OAS1* gene variant are associated with poor outcomes of COVID-19 infection
Management of AD during COVID-19 pandemic	Modification of care strategies in AD	1. The COVID-19 pandemic profoundly changes the way of AD management 2. Telemedicine is feasible and well accepted in assessing and managing AD during the COVID-19 pandemic
	Potential impact of drug therapies for AD on COVID-19	1. Cholinesterase inhibitors therapies have not been reported on reducing the infection rate and mortality of COVID-19 thus far 2. Prescribing ARBs but not ACEIs is significantly associated with a lower risk of COVID-19 occurrence among AD patients 3. The impact of CCB usage upon the efficacy of COVID-19 in AD patients remains to be clarified
	Effect of COVID-19 vaccines on AD	1. Vaccinated AD patients are still at increased risk for COVID-19 breakthrough infection 2. AD patients may be vulnerable to delirium after taking the COVID-19 vaccine 3. Accelerated focal amyloid-β deposition induced by low-level inflammation after COVID-19 vaccination in AD patients 4. The combination of anti-amyloid-β immunotherapies and adenoviral COVID-19 vaccines may increase the risk of cerebral hemorrhage in patients with AD
Mechanisms of the link between COVID-19 and AD		1. Inflammation, aging, insulin resistance, acetylcholine, and amyloid-β might mediate the mechanistic links between COVID-19 and AD 2. Risk alleles of *APOE* and *OAS1* are associated with both AD and poor COVID-19 outcomes 3. Dysregulated immunity may play a key role in the mechanistic link between COVID-19 and AD

*ACEI* Angiotensin converting enzyme inhibitor; *AD* Alzheimer’s disease; *APOE* Apolipoprotein E; *ARB* Angiotensin II receptor blockers; *CCB* Calcium channel blocker; *COVID-19* Coronavirus disease 2019; *OAS1* Oligoadenylate synthetase 1

### Impact of COVID-19 on AD

#### Effect on dementia severity

Recent studies have revealed pronounced systemic inflammation and cytokine storm in severe COVID-19 [103]. Accumulating evidence from post-mortem studies suggests that COVID-19-related neuropathological alterations are most likely to be immune-mediated [86, 104–106]. Severe COVID-19 and prolonged hypoxia would exacerbate the severity of pre-existing cognitive impairment of AD. This further affects the quality of life of AD patients. A most recent case report also suggested that pre-symptomatic people without a diagnosis of AD experience an acceleration of cognitive decline due to prolonged hypoxia related to COVID-19 [107].

Furthermore, previous experimental data suggested that isolation aggravates cognitive impairment in AD animal models [108–110]. Thus, it raised the question of whether restriction measures implemented to limit the rapid spread of the SARS-CoV-2 virus during the COVID-19 pandemic would exacerbate the cognitive deficits of AD patients. Barguilla et al. found that 60% of AD patients suffered from cognitive decline during 2 months of lockdown [6]. Similarly, a nationwide survey in Italy interviewing caregivers of 4913 patients with dementia reported acute worsening of cognitive functions in 55.1% of patients during COVID-19 quarantine, mainly in subjects with AD and dementia with Lewy bodies [111]. Interestingly, pre-existing physical independence in motor function was associated with cognitive worsening (OR, 1.85; 95% CI 1.42–2.39), whereas pandemic awareness was a protective factor against worsening of cognitive symptoms (OR, 0.74; 95% CI 0.65–0.85). In addition, a study in France showed that worsened cognitive function was only present in AD patients who had definite neuropsychiatric changes during a period of 2-month isolation, accounting for 26% of the patients [5]. These observations support that the COVID-19 pandemic exerts a negative impact on the cognitive function of AD.

#### Effect on neuropsychiatric symptoms

Neuropsychiatric manifestations are a common feature in dementia patients, affecting 80% of patients over the course of the disease. Accumulating evidence shows that 36%–78% of individuals hospitalized with COVID-19 display neurological symptoms including neuropsychiatric manifestations [112]. On the other hand, it is also reported that neuropsychiatric symptoms may emerge as the initial presentation of COVID-19 in subjects with dementia. Bianchetti et al. summarized the clinical manifestations of COVID-19 in dementia patients [113]. Among 82 patients diagnosed with dementia, the most common initial symptoms of COVID-19 were delirium (67%) and worsening of the functional status (56.1%). On the contrary, typical symptoms of COVID-19, including fever (47.6%), dyspnea (43.6%), and cough (13.4%), were less common in dementia patients [113]. It is worth noting that as many as 50% of the delirium symptoms manifest in the hypoactive form. These results suggested that the clinical presentation of COVID-19 in patients with dementia is atypical, and SARS-CoV-2 infection could be considered in the presence of manifestation and/or exacerbation of neuropsychiatric symptoms during this pandemic. Early recognition and treatment are important for preventing development of severe consequences of COVID-19 in dementia patients.

Neuropsychiatric symptoms tend to deteriorate under external stressors. Numerous cross-sectional studies in different countries have investigated the impact of the COVID-19 pandemic and social-distancing measures on neuropsychiatric symptoms of AD. They consistently demonstrated that the COVID-19 pandemic and related restrictions lead to the worsening of neuropsychiatric symptoms in AD patients [5, 7, 114, 115], with most pronounced neuropsychiatric symptoms being agitation, anxiety, and depression. The worsening of irritability, sleep disturbance, apathy, aberrant motor activity, appetite disturbances, and delusion were also reported in some of these studies. Bretonniere et al. found that the duration of confinement and increased stress of caregivers significantly correlate with the severity of neuropsychiatric symptoms [5]. Besides, limited understanding of the COVID-19 situation and lack of outpatient rehabilitation services are also associated with a deterioration of neuropsychiatric symptoms [114, 116]. Apart from the aforementioned studies completed after 1–2 months of COVID-19 lockdown, Chen et al. reported a longitudinal 1-year follow-up study comparing cognitive function of AD patients before and after COVID-19 lockdown to determine the long-term impact of confinement on AD patients. They found significantly worsening of neuropsychiatric inventory at 1-year follow-up compared to baseline, and neuropsychiatric symptoms deteriorated in 43.8% of AD patients. Regression analysis implicated that the decline of social contact and sleep disturbance at baseline contributed to the worsening of neuropsychiatric symptoms in AD patients [7]. Muntsant et al. examined the impact of long-term isolation in male 3×Tg-AD mice that model advanced stages of AD, compared to age-matched counterparts with normal aging [117]. The isolated 3×Tg-AD mice displayed exacerbated hyperactivity, emergence of bizarre behaviors, and re-structured anxiety-like patterns and coping-with-stress strategies. This study further confirmed that social isolation exacerbates neuropsychiatric symptoms in AD.

Taken together, the worsening of neuropsychiatric disturbances in AD is not only a direct result from SARS-CoV-2 infection, but also secondary to pandemic-related socioeconomic changes.

#### Effect on disease progression

Unlike consistent deterioration of neuropsychiatric symptoms in different reports, there is no consensus on the impact of the COVID-19 pandemic and related restrictions on dementia progression in AD patients. Gan et al. completed a longitudinal follow-up study in 131 AD patients experiencing confinement for a mean duration of 8.89 months to determine the impact of implemented restrictions during the COVID-19 pandemic in AD patients [118]. They found that over 50% of these patients presented significant cognitive decline at an average of 14.07-month follow-up compared to baseline, as assessed by the Chinese Mini-Mental State Examination and Montreal Cognitive Assessment (MoCA). However, the extent of cognitive decline of these participants was similar to that of strictly matched 131 AD patients as controls, who were followed up before the COVID-19 pandemic. These results suggested that cognitive decline during the pandemic in AD patients resulted from the intrinsic neurodegenerative process rather than the confinement. These data are consistent with another longitudinal 1-year follow-up study [7], which reported that the decline of MMSE scores in AD patients during the pandemic was similar to the rate of yearly decline in previous studies before the pandemic [7]. On the contrary, Tsiakiri et al. longitudinally assessed the cognitive performance of 21 AD patients during 7-month follow-up that corresponded to the strict lockdown period in Greece [115]. They found significantly greater worsening of MMSE and MoCA scores in AD patients during the COVID-19 pandemic lockdown compared to the AD controls who completed follow-up before the lockdown. This suggested that COVID-19 pandemic aggravates the rate of cognitive decline in AD. Therefore, longer follow-up is crucial to evaluate the direct impact of the COVID-19 pandemic on the progression of AD. Animal model studies can provide hints and evidence for potential mechanisms underlying isolation and AD. Previous studies in Tg2576, APP/PS1, and 5×FAD transgenic mice have reported that isolation leads to acceleration of AD pathology manifested as an increase in amyloid-β disposition in the brain [108–110]. A recent study reported hippocampal atrophy and left–right asymmetry in isolated male 3×Tg-AD mice compared to age-matched counterparts with normal aging, although there was no exacerbation of tau pathology in the hippocampus [117]. Unfortunately, there is no longitudinal amyloid-related imaging or cerebral glucose metabolism imaging study in patients with AD to assess whether COVID-19 aggravates the amyloid burden in the brain. From the translational perspective, it is of great importance in the future to longitudinally follow up neuropsychological and neuroimaging performance in community-based AD cohorts to determine the long-term effect of COVID-19 and related restrictions on dementia progression and AD-related mortality.

#### Trigger for AD

Neurological symptoms including memory loss are reported in up to 30% of COVID-19 cases [112, 119]. Several structural neurologic abnormalities can persist for a long time after acute COVID-19 infection, which is part of the long COVID spectrum [120–122]. A recent international cohort study revealed that over 70% of COVID-19 patients experienced cognitive dysfunction and about 30% of COVID-19 patients have long-term memory issues at 7 months after infection [121]. It has been proven that SARS-CoV-2 infection not only induces innate and adaptive immune activation in multiple peripheral organs but also disrupts the BBB and alters the inflammatory factor profile of the brain [86, 103, 123]. Brain autopsies on COVID-19 patients have shown sparse SARS-CoV-2 virus in cortical neurons, accompanied by infiltration of minimal immune cells [106]. In line with this observation, an experimental study ascertained that SARS-CoV-2 infection induces neuronal death in mice overexpressing human ACE2 receptors [106]. Strikingly, the latest breakthrough came from the study by Charnley and colleagues, which identified two short peptides from the SARS-CoV-2 proteome that self-assemble into amyloid assemblies [124]. Furthermore, these protease-resistant amyloids were shown to be highly toxic to neuronal cells, which shared similarities to the toxic amyloid-β in AD. These findings raise the possibility that SARS-CoV-2 infection could induce long-term neurodegeneration. Data from brain lysates of COVID-19 patients revealed activation of tau hyperphosphorylation-associated signaling pathways, suggesting that AD-like features are involved in COVID-19 neuropathology [125]. Serum neurodegenerative biomarkers (total tau, phosphorylated tau-181, glial fibrillary acidic protein, neurofilament light chain) from hospitalized COVID-19 patients without dementia are elevated to levels observed in AD, and are associated with encephalopathy and worse outcomes [126]. Another recent study has shown that elevated concentrations of neuroaxonal damage markers (14-3-3 protein and neurofilament light chain) in the cerebral spinal fluid (CSF) could predict the severity of neurologic disability at 18 months of follow-up [127]. These lines of evidence support the role of SARS-CoV-2 infection in triggering future AD dementia.

Most recent metabolic brain imaging studies showed that acute SARS-CoV-2 infection could induce hypometabolism of a widespread cerebral network including the frontoparietal and temporal cortex, insula, and basal ganglia. At the 6-month follow-up, however, brain hypometabolism had partially or totally recovered, although several attention deficits and executive dysfunctions but not memory problems remained [104, 128, 129]. Considering the relatively small sample size and short follow-up, these results should be interpreted with caution. Strikingly, Douaud and colleagues reported the first and largest longitudinal imaging study comparing brain MRI scans of 401 individuals before and after SARS-CoV-2 infection to scans of 384 well-matched controls [130]. The average interval between their COVID-19 diagnosis and the second scan was 141 days. Compared to the control group, the SARS-CoV-2-positive participants showed a greater reduction of grey matter thickness and tissue-contrast in the orbitofrontal cortex and parahippocampal gyrus and greater changes in markers of tissue damage in regions functionally connected to the primary olfactory cortex. These results revealed a consistent spatial pattern of longitudinal abnormalities in limbic brain regions, which are the main part of the olfactory network, following SARS-CoV-2 infection. In addition, the SARS-CoV-2-positive participants showed a greater reduction of global brain size and significant worsening of executive function measured by the Trail Making Test. However, neither signs of memory impairment when comparing the two groups, nor any association of the parahippocampal gyrus and other memory-related regions with the selected cognitive tests was detected. It remains to be determined whether the loss of grey matter and tissue damage of those specific limbic regions may in turn increase the risk of memory deficits and dementia in the longer term. More recently, Xu and colleagues estimated the risks and burdens of incident neurologic disorders at 12 months following acute SARS-CoV-2 infection in a large-scale cohort that included 154,068 individuals with COVID-19 [131]. Increased risks of memory problems (HR, 1.77; 95% CI 1.68–1.85) and AD (HR, 2.03; 95% CI 1.79–2.31) were shown at 12 months following acute SARS-CoV-2 infection. It should be mentioned that the abovementioned data were acquired before the Omicron variant has become dominant in most countries. Recent evidence suggests attenuated pathogenicity of the Omicron variant in comparison to the wild-type strain and the Alpha, Beta and Delta variants [132–135]. Accordingly, the latest report from the UK COVID Symptom Study also confirmed a reduced odds of long COVID symptoms between 0.24 and 0.50 for the Omicron variants compared with the Delta variants [136]. Thus, different SARS-CoV-2 variants may be associated with different risks of developing AD. It is also the case for patients vaccinated or not. The clinical impacts of different SARS-CoV-2 variants on triggering of AD have not been clarified.

In addition to the possible direct effect of SARS-CoV-2 infection on the development of dementia, previous studies suggested that social isolation and loneliness are related to cognitive decline and the risk of future dementia [137]. In particular, a recent study by Shen and colleagues utilizing a UK-Biobank cohort of 462,619 participants with a mean follow-up period of 11.7 years indicated that social isolation is associated with a 1.26-fold increase of risk of developing dementia, which is independent of loneliness and other risk factors [138]. Furthermore, structural MRI and transcriptomic data illustrated that socially isolated individuals have lower gray matter volumes in temporal, frontal, and hippocampal regions coupled with different molecular functions. These findings have clinical implications in the context of the COVID-19 pandemic, suggesting that quarantine for the COVID-19 pandemic may exert a pernicious effect on cognition later in life. Thus, social relationship interventions should be targeted to alleviate the long-term impact of both social isolation and loneliness on cognitive outcomes.

The longer-term effect of COVID-19 infection and related quarantine on the risk of developing AD will be one of the priority areas for future research. Longitudinal studies of brain structure and function with longer follow-up intervals are warranted, and will provide guidance for care strategies after acute COVID-19 and public health policies.

### Impact of AD on COVID-19

#### Susceptibility to COVID-19 infection

There is growing evidence supporting that AD patients are at increased risk of COVID-19 infection. A retrospective case–control analysis of 61.9 million adult and senior COVID-19 patients in the US before August 21, 2020 unraveled that AD patients had a higher risk of COVID-19 compared to patients without dementia (adjusted OR, 1.86; 95% CI 1.77–1.96) [139]. Interestingly, the study also revealed that Blacks with dementia had a higher risk of COVID-19 than Whites (adjusted OR, 2.86; 95% CI 2.67–3.06). More recently, another large-scale study including 436,823 subjects (≥ 50 years old and < 90 years old) from the US has also shown that patients with AD are associated with higher odds of being diagnosed with COVID-19 than patients without AD (OR, 1.688; 95% CI 1.558–1.828) [140]. In accordance with these observations in the US, data from the UK Biobank cohort revealed that a diagnosis of AD is strongly associated with SARS-CoV-2 infectivity, with AD patients showing greater susceptibility to SARS-CoV-2 infectivity compared to individuals without AD (OR, 4.15; 95% CI 3.22–5.34) [141]. However, nationwide data from Korea reported that the positive rate of COVID-19 testing did not differ between individuals with and without AD, raising doubts on the association of AD with increased susceptibility to COVID-19 [142]. Further investigations from more countries are needed to clarify the issue. In addition to the intrinsic link between AD and COVID-19, socioeconomic factors should not be underestimated in explaining the possible increased risk of COVID-19 in AD patients. Cognitive impairment and neuropsychiatric symptoms make it challenging for AD patients to understand and comply with safeguarding procedures, such as wearing masks and maintaining appropriate physical distancing. Ignoring or forgetting warnings and an inability to follow self-quarantine measures also increase the risk of infection [143]. Furthermore, most of the individuals with AD live in institutional settings (nursing or care homes), where rates of infection are disproportionately high. This situation facilitates rapid transmission of the SARS-CoV-2 virus and increases the risk of infection among AD patients. In summary, biological and socioeconomic factors work together to make individuals with AD vulnerable to SARS-CoV-2 infection.

#### Prognosis of COVID-19 infection

To date, it has become clear that AD is a strong risk factor for increased mortality of COVID-19. Bianchetti et al. assessed COVID-19-related mortality in dementia among 627 subjects hospitalized for COVID-19 [113]. They found a significantly higher mortality in patients with dementia compared to those without dementia (62.5% vs 26.2%). Regression analysis revealed that dementia is an independent risk factor for COVID-19-related mortality with an OR of 1.84 (95% CI 1.09–3.13). Covino et al. reported risk factors for COVID-19-related mortality in 69 symptomatic COVID-19 patients aged ≥ 80 years [144]. Interestingly, in this population with advanced age, the risk of death was not age-dependent whereas severe dementia was an independent risk factor for death (OR, 3.87; 95% CI 1.23–12.17). These data indicate that the pre-existing dementia exacerbates the severity and mortality of COVID-19. More recently, a retrospective study including 61.9 million adult and senior COVID-19 patients from the US reported that the 6-month hospitalization risk for adult and senior patients with AD and COVID-19 (61.54%) was significantly higher than that for adult and senior patients with COVID-19 but without dementia (23.26%) or with AD but without COVID-19 (13.80%) [139]. Additionally, the 6-month mortality risk for adult and senior patients with AD and COVID-19 (19.23%) was significantly higher than that for adult and senior patients with COVID-19 but without dementia (4.81%) or with AD but without COVID-19 (9.71%). Consistently, latest analysis of a national healthcare database in the US has shown that patients with AD have increased odds of hospitalization (OR, 1.428; 95% CI 1.139–1.791) and death (OR, 1.695; 95% CI 1.383–2.078) compared to patients without AD [140]. Similarly, two large-scale UK Biobank cohort studies consistently identified pre-existing dementia as the highest risk factor for COVID-19 hospitalization and mortality [14, 141]. Subgroup analysis from one of the studies revealed that diagnoses of AD predict an increased risk of COVID-19 death with an odds ratio of 4.17 (95% CI 2.87–6.05) [141]. In line with these results, another nationwide cohort study from Korea reported that AD is associated with both an increased risk of severe COVID-19 complications (OR, 2.25; 95% CI 1.54–3.28) and an increased risk of mortality (OR, 3.09; 95% CI 2.00–4.78) [142]. Additionally, a systematic review of 9 studies has shown that the mortality rate of individuals with dementia after being infected with COVID-19 is higher than that of those without dementia (OR, 5.17; 95% CI 2.31–11.59) [145]. Taken together, a diagnosis of AD is undoubtfully associated with increased mortality of COVID-19. Close and tailored monitoring of patients with AD is needed to reduce the impact of COVID-19 on this frail population.

Several studies have explored the risk factors for the occurrence and severe clinical outcomes of COVID-19 in dementia patients. In a systematic review of 10 studies, the association between dementia and mortality of COVID-19 is influenced by age and hypertension [146]. Additionally, a considerable body of evidence indicates that *APOE* ε4 allele, a well-known AD risk factor, increases the risk of severity and mortality of COVID-19, independent of pre-existing dementia, cardiovascular disease, and type-2 diabetes [147–149]. In the UK Biobank community cohort study, the risk of COVID-19-related hospitalization was more than two-fold higher among *APOE* ε4/ε4 homozygotes compared to ε3/ε3 homozygotes [149]. Similarly, homozygous *APOE* ε4/ε4 patients exhibit a more than two-fold increased hazard ratio for death relative to *APOE* ε3/ε3 homozygous patients [148]. Strikingly, oligoadenylate synthetase 1 (*OAS1*), a newly reported risk gene for AD, was recently identified as a putative risk gene associated with severe COVID-19 in intensive care patients in human genome-wide association studies [150, 151]. These findings suggest the importance of investigating molecular pathways involved in the link between AD and severe COVID-19 infection, as well as understanding the risk factors associated with AD to reduce the occurrence and severe clinical outcomes of COVID-19.

### Management of AD during the COVID-19 pandemic

#### Modification of care strategies for AD

The isolation and quarantine measures during the COVID-19 pandemic have a profound impact on the management strategies for AD. Telemedicine services have been advocated and developed rapidly during the COVID-19 pandemic. Several studies before the COVID-19 pandemic have demonstrated comparable accuracy and reliability of diagnosis and monitoring, as well as comparable visit satisfactions between telemedicine services and face-to-face clinical evaluation [152, 153]. Moreover, studies have revealed a similar effect on the annual changes of MMSE scores between patients using the telemedicine services and patients attending the dementia clinic in person [154]. Strikingly, a large-scale randomized trial found that providing care monthly to patients with dementia and caregivers via telephone and Internet improved patient’s quality of life after 12 months in comparison to standard care [155]. In the context of COVID-19, telemedicine and digital devices, including telephones and video conferences, also benefit remote monitoring and management of patients with dementia [156–164]. As mentioned above, restriction measures during the COVID-19 pandemic have a detrimental impact on neuropsychiatric symptoms. Telemedicine decreases the frequency and intensity of neuropsychiatric symptoms, as well as improving the caregiver's well-being and mental health in a systematic review of 22 studies [165]. Tele-rehabilitation platforms for neurorehabilitation care (e.g., fitness training and cognitive training) with remote supervision have a positive effect on patients and reduces the burden on family caregivers [166–168]. However, barriers still exist in the practice of telemedicine services, such as issues of patient privacy, confidentiality and security of information, limitations in clinical data acquisition, lack of technological literacy, and connectivity problems [153, 162, 163, 169]. Most patients with AD are older adults who may have greater difficulty accessing technological devices or Internet services. Therefore, support from caregivers to facilitate the virtual visit is essential to the success of direct-to-home care for dementia [170]. Although telemedicine services are feasible and well accepted in assessing and managing AD, whether they could improve clinical outcomes in patients with AD remains to be clarified.

#### Potential impact of drug therapies for AD on COVID-19

Cholinesterase inhibitors increase the availability of acetylcholine at synapses in the brain and are one of the few drug therapies that have been proven clinically useful in the treatment of AD. Currently available FDA-approved cholinesterase inhibitors for the treatment of AD are donepezil, rivastigmine, and galantamine. On the other hand, it is hypothesized that dysfunctions of the nicotinic cholinergic system may be involved in severe COVID-19, which contribute to uncontrolled cytokine storm [171]. Acetylcholine stimulation of α7 nicotinic acetylcholine receptor (α7-nAChR) on peripheral macrophages has been proven to suppress pro-inflammatory cytokine secretion [172]. Treatment strategies targeting the cholinergic system are proposed to induce symptomatic improvement in both AD and COVID-19. However, no studies have reported if cholinesterase inhibitor therapies could reduce the infection rate and mortality of COVID-19.

Hypertension is a common comorbidity and risk factor for both AD and COVID-19. Accumulating evidence has indicated the beneficial effects of long-term use of antihypertension medications on decreasing the risk of developing AD, including angiotensin II receptor blockers (ARBs) and angiotensin converting enzyme inhibitors (ACEIs) [173, 174]. By retrospective analysis of 436,823 patients with SARS-CoV-2 infection, Wang and colleagues found that prescribing ARBs but not ACEIs is significantly associated with a lower risk of COVID-19 occurrence among AD patients [140]. Dysregulation of calcium ion (Ca^2+^) hemostasis plays an important role in the pathogenesis of AD. Calcium channel blocker (CCB) nimodipine is a treatment choice for AD. Based on the fact that Ca^2+^ is associated with SARS-CoV-2 virus entry into host cells and the inhibitory effect of CCBs on infections of several other viruses, CCBs are postulated as a potential therapeutic strategy for the management of COVID-19 [175, 176]. Unfortunately, it is still controversial whether the use of CCBs could improve the outcomes of COVID-19 in clinical practice. In a large cohort study including 64,781 patients with COVID-19 in the US, Rosenthal et al. found that the use of CCB is independently associated with decreased in-hospital mortality (OR, 0.73; 95% CI 0.68–0.79) [177]. However, several large-scale retrospective studies have implicated that CCBs are not associated with an increased risk of COVID-19 diagnosis or severity [178–180]. A meta-analysis study including 31 studies also indicated no influence of CCBs on reduced mortality of COVID-19 (OR, 1.21; 95% CI 0.98–1.49), whereas subgroup analysis showed that CCBs are associated with a decreased mortality in hypertensive COVID-19 patients (OR, 0.69; 95% CI 0.52–0.91) [181]. In the latest cohort study including 245 hospitalized patients with hypertensive COVID-19, however, Mendez and colleagues found that dihydropyridine CCBs increase the risk of COVID-19-related intubation or death compared to patients not taking dihydropyridine CCBs [182]. Therefore, prospective randomized trials are required to explore potential effective treatments for both AD and COVID-19.

#### Effect of COVID-19 vaccines on AD

Vaccination is now regarded as the most effective countermeasure to prevent the spread of COVID-19. Large-scale worldwide COVID-19 vaccination programs are being rapidly implemented, whereas vaccinated AD patients are still at increased risk for COVID-19 breakthrough infection. A retrospective cohort study of 262,847 vaccinated older adults in the US has shown that the overall risk of breakthrough infections beginning 14 days following vaccination in AD was significantly higher than that in older adults without dementia between December 2020 and August 2021 (10.3% vs 5.6%; adjusted OR, 1.53; 95% CI 1.22–1.92) [183]. The overall risk for hospitalization after breakthrough infections was 39.5% in AD patients in contrast to 1.5% in AD patients who had no breakthrough infections (HR, 54.1; 95% CI 34.0–86.0). Interestingly, fully vaccinated women with AD had a lower risk of breakthrough infections than men after matching for demographic factors and comorbidities (adjusted OR, 0.68; 95% CI 0.47–0.98) [183]. Thus, it is of great necessity to continuously monitor breakthrough SARS-CoV-2 infections and outcomes in vaccinated patients with AD. Meanwhile, post-vaccination adverse events have been reported. A patient with AD was reported to manifest delirium following the first dose of vaccination with an inactivated COVID-19 vaccine [184]. This suggests that individuals with AD may be vulnerable to delirium after receiving the COVID-19 vaccine. ^18^F-Florbetaben PET scan in a patient diagnosed with AD demonstrated ill-defined subcutaneous uptake on the vaccination site and focal uptake next to an ipsilateral axillary lymph node after administration of the first dose of mRNA COVID-19 vaccine. This suggests possible accelerated amyloid-β deposition induced by low-level inflammation after COVID-19 vaccination in AD patients [185]. However, whether COVID-19 vaccination exacerbates amyloid-β immune reactivity in the brain is still unknown and needs to be clarified in the future.

Recently, SARS-CoV-2 vaccine-induced immune thrombocytopenia and thrombosis (VITT) have been identified as a rare adverse effect of adenoviral vector COVID-19 vaccines with an incidence rate of 1.33% (95% CI 1.19 to 1.47 at 8–14 days) [186]. A prospective, hospital-based study by Sue and colleagues showed that VITT usually develops between 5 and 48 days after the first dose of the vaccine with an overall mortality rate of 22% [187]. Palaiodimou and colleagues reported that 51% of patients with VITT present with cerebral venous sinus thrombosis (CVST) (95% CI 36%–66%) [188]. VITT is independently associated with a higher risk of CVST when compared to patients without VITT with thrombotic events after vaccination (OR, 13.8; 95% CI 2.0–97.3). Intracranial hemorrhage is a common presentation in patients with postvaccination CVST and concomitant VITT, occurring in 36%–68% of cases [187, 189]. The presence of intracranial hemorrhage is an independent risk factor for death (OR, 4.544; 95% CI 2.188–9.437) [187]. Available evidence suggests that anti-platelet factor-4 autoimmune antibodies are involved in the pathogenesis of VITT. In light of AD, amyloid-β-targeting monoclonal antibody immunotherapies have emerged as a promising but highly arguable treatment for AD. Data from large-scale clinical trials have revealed that 10%–30% of AD patients experience amyloid-related imaging abnormalities-hemosiderin deposition (ARIA-H) (cerebral microhemorrhages, cerebral microhemorrhages, and superficial siderosis) after receiving different anti-amyloid-β immunotherapies [190–195]. Existing data suggest that antibody-mediated amyloid-β deposition in vessels and perivascular inflammation contribute to ARIA-H [196]. Given the common occurrence of cerebral amyloid angiopathy in AD, elderly AD patients with amyloid-β-targeting treatment may have an increased risk of intracranial hemorrhage. Despite diverse immune mechanisms underlying intracranial hemorrhage triggered by VITT and anti-amyloid-β immunotherapies, cautions should be paid to administration of adenoviral COVID-19 vaccine in AD patients receiving treatment with anti-amyloid-β antibody [197]. Therefore, it is of great importance to accurately evaluate comorbidities and risk factors in the frail AD population before COVID-19 vaccination.

### Mechanisms of the link between COVID-19 and AD

#### Neuroinflammation

**Fig. 2:**
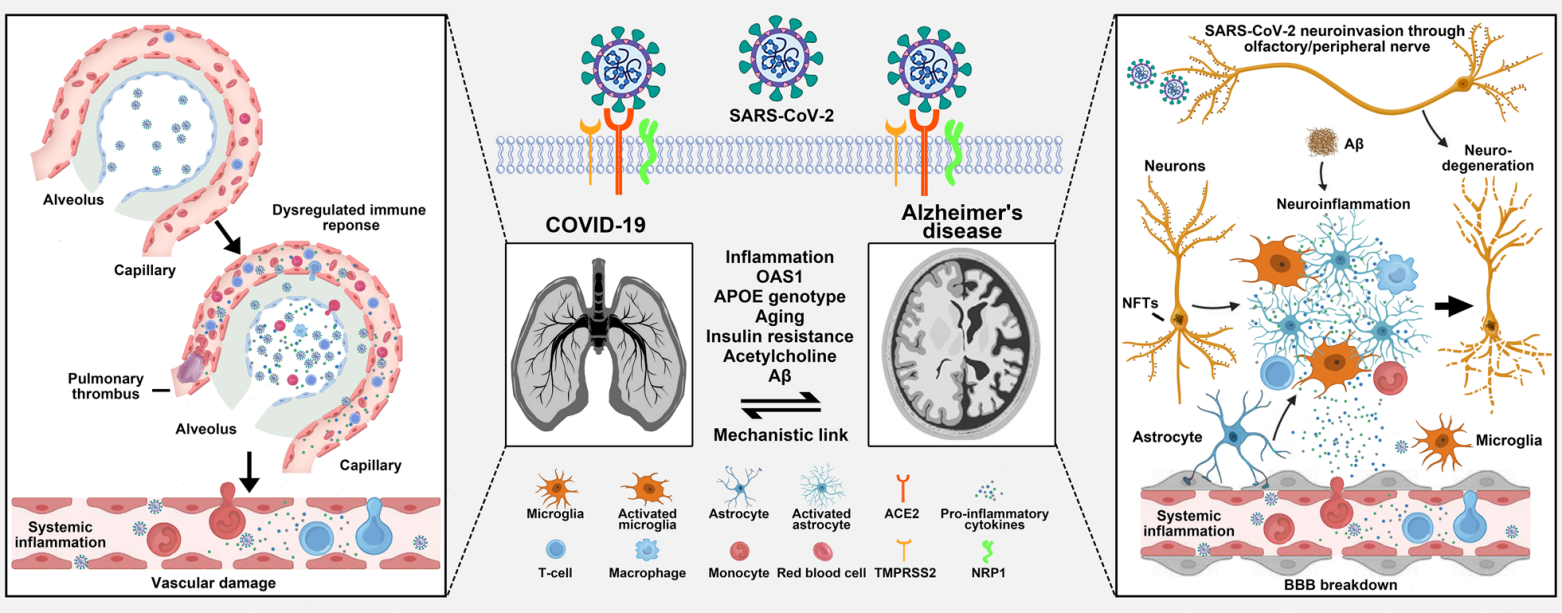
Putative mechanisms of the link between COVID-19 and Alzheimer's disease (AD). With the aid of TMPRSS2, the S protein of SARS-CoV-2 binds with ACE2 to enter the host cell. The SARS-CoV-2 virus may affect the brain in the following ways: vascular damage (BBB breakdown), systemic inflammation, and direct neuroinvasion. Neuroinflammation, OAS1, *APOE* genotype, aging, insulin resistance, acetylcholine, and Aβ may mediate the links between COVID-19 and AD. ACE2: angiotensin-converting enzyme 2; Aβ: Amyloid-beta; APOE: apolipoprotein E; BBB: blood–brain barrier; COVID-19: Coronavirus disease 2019; NFTs: neurofibrillary tangles; NRP1: neuropilin 1; OAS1: oligoadenylate synthetase 1; SARS-CoV-2: severe acute respiratory syndrome coronavirus 2; TMPRSS2: transmembrane serine protease 2

Accumulating evidence supports a mechanistic link between AD and COVID-19 (Fig. 2). It has been demonstrated that SARS-CoV-2 virus could enter the brain, although the routes remain controversial [13, 198]. However, very low levels of the SARS-CoV-2 virus are detected in the brains of patients dying from COVID-19 [86, 104–106]. Consistent with the post-mortem findings, CSF data from COVID-19 patients with neurological symptoms illustrated that direct infection of the CNS with SARS-CoV-2 seems to be rare [199]. Strikingly, prominent microglial activation in the white matter of the brainstem, hippocampus and cerebellum is observed in post-mortem studies of COVID-19 patients, along with accumulations of parenchymal cytotoxic T lymphocytes. These observations suggested that COVID-19-related neuropathological alterations are not directly induced by the presence of SARS-CoV-2 in the brain, but are most likely mediated by the neuroimmune system. The latest data on the CSF profile indicated that the blood-CSF barrier disruption in the absence of intrathecal inflammation is a cardinal feature of COVID-19 patients with neurological involvement, compatible with cerebrospinal endotheliopathy [127, 199]. These results revealed that the peripheral SARS-CoV-2 virus imposes an adverse impact on the CNS through disruption of the blood-CSF barrier, regardless of manifestation of CNS symptoms. A large-scale single-cell transcriptome atlas revealed that severe COVID-19 induces pronounced systemic inflammation and cytokine storm [103]. Serum pro-inflammatory cytokines, including IL-6, TNF-α, IL-1β, and IFN-γ, are dramatically elevated in critical COVID-19 patients and identified as markers of poor prognosis. Hyperactivation of NLRP3 inflammasomes in macrophage lineage cells mediates the release of pro-inflammatory cytokines, which contributes to the uncontrolled systemic immune response and promotes the development of severe COVID-19 [90, 200]. On the other hand, it is well-known that neuroinflammation is a key component of AD pathogenesis [201]. There is a complicated cross-talk between the peripheral and central immune systems in AD [202]. Serum IL-6 level has also been reported to be significantly higher in AD patients than in healthy controls [203]. The NLRP3 inflammasome is the most abundant inflammasome in the CNS and one of the key contributors to neuroinflammation in AD [204]. Hyperactivation of the NLRP3 inflammasome has also been proven to inhibit the phagocytosis of amyloid-β by microglia, and exacerbate amyloid-β deposition and tau pathology, which accelerate the initiation and development of AD pathology [205, 206]. These findings support that dysregulation of the immune signaling pathway could be a potential mechanism for the link between COVID-19 and AD. Systemic immune abnormality in AD may promote pro-inflammatory cytokine release and tissue damage under SARS-CoV-2 infection, which contribute to the critical illness of COVID-19. Recently, Magusali and colleagues provided new evidence for a genetic link between the risk of AD and critical outcomes of COVID-19 from the perspective of innate immune system in the brain. Their study identified single nucleotide polymorphisms of the OAS1 gene to be associated with both increased risk of AD and predisposition to severe COVID-19 [207]. They found that OAS1 is required to limit the pro-inflammatory response of myeloid cells induced by IFN-γ stimulation. On the contrary, *OAS1* risk alleles for AD and severe COVID-19 are all linked with decreased OAS1 expression, which exaggerates the production of TNF-α with IFN-γ stimulation. These intersections between AD and COVID-19 point to a crucial role of regulating interferon pathways in the treatment of both diseases.

#### *APOE* ε4 status

The impact of *APOE* variants on SARS-CoV-2 infection has been a focus in studies on the mechanistic link between AD and COVID-19. The ε4 allele of *APOE* is the strongest genetic risk factor for developing AD [208]. Recent studies also reported that *APOE* has an impact on COVID-19 susceptibility and severity. *APOE* ε4 carriers exhibit an increased SARS-CoV-2 infection rate and mortality compared to non-carriers [147–149]. COVID-19 patients carrying *APOE* ε4 also show a significantly higher level of serum pro-inflammatory cytokines than patients with the *APOE* ε3/ε3 genotype [209]. Xiong et al. used a pathway analysis approach and identified *APOE4* as an important risk factor for the severity of COVID-19 in patients with AD [210]. In human induced pluripotent stem cell (iPSC)-derived neurons and astrocytes, Wang and colleagues found that *APOE* ε4/ε4 genotype induces an increased rate of SARS-CoV-2 infection than the *APOE* ε3/ε3 genotype. Moreover,* APOE4* astrocytes exhibited a more severe response following SARS-CoV-2 infection. This study provides the first insight into a possible *APOE*-mediated mechanism of COVID-19 vulnerability and severity [211]. The latest report by Ostendorf and colleagues further confirmed the influence of *APOE* variants on COVID-19 outcomes in an established SARS-CoV-2-infected *APOE* knock-in mouse model [148]. They demonstrated that *APOE4* and *APOE2* mice exhibit increased SARS-CoV-2 viral loads and insufficient antiviral immune responses early after infection compared to *APOE3* mice. These findings highlight that the *APOE4*-mediated poor outcomes of COVID-19 are closely related to its effect on the host immune response. Zhang and colleagues found that although both APOE3 and APOE4 reduce ACE2-mediated Spike docking onto the cell surface by directly interacting with the ACE2 receptor, APOE4 indeed inhibits SARS-CoV-2 viral entry to a lesser extent compared to APOE3. They postulated that this disparity may be due to the more compact structure of APOE4 and its smaller spatial obstacle to compete against Spike protein binding to the ACE2 receptor [209]. Pathway studies are required to further elucidate the molecular basis underlying *APOE4*-associated regulation of the vulnerability to and severity of COVID-19. Prospective studies are also warranted to evaluate *APOE* variants as a biomarker for identifying patients at high risk of poor outcomes of COVID-19.

#### Aging

Aging is another compelling intersection between AD and COVID-19 [13]. AD is the most common neurodegenerative disease affecting aged people. Cellular senescence disturbs multiple homeostatic processes in the CNS, inducing chronic inflammation, BBB breakdown, amyloid deposits, and tau pathology, which in turn contribute to the pathogenesis of AD [212, 213]. Advanced age is also a well-recognized risk factor for developing serious illness and death from COVID-19. Previous data from the UK Biobank cohort showed that individuals aged ≥ 80 years have a significantly higher risk for COVID-19 hospitalization compared to the 65–69 age group (OR, 2.02; 95% CI 1.41–2.89) [14]. The mortality rate of COVID-19 patients aged 80 years or older is more than 20% [214]. It is also the case for the dementia population. The latest study focusing on vaccinated COVID-19 patients with dementia in the US found that older individuals with AD (age ≥ 80 years) have higher risks for breakthrough infections than younger patients with AD (age 65 to 79 years) (adjusted OR, 1.56; 95% CI 1.09–2.22) [183]. Aging-related factors, including immune surveillance impairment, chronic inflammation, and reactive oxygen species accumulation, are suggested to be associated with greater severity and mortality in aged COVID-19 patients [215]. Thus, aging-mediated mechanisms could be shared by both AD and COVID-19, although the key intersections between AD and COVID-19 in aging-related pathways remain to be determined.

#### Insulin resistance

Emerging evidence from human and animal studies supports that altered brain insulin resistance is also an important pathological hallmark of AD [216], and regarded as one of the molecular links between AD and diabetes mellitus. Dysregulation of the innate immune system and cytokine-mediated chronic low-grade inflammation induced by insulin resistance in the periphery has been well understood [217]. The pre-existing insulin resistance-induced chronic inflammation would facilitate augmentation of systemic immune response after SARS-CoV-2 infection, which significantly increases the risk of critical illness of COVID-19. Moreover, insulin resistance-related comorbidities, including obesity, diabetes mellitus, and hypertension, are highly prevalent, which implicate a higher risk of developing AD and severe COVID-19 [218, 219]. However, the precise mechanisms of increased COVID-19 susceptibility and severity in AD in the presence of insulin resistance are still poorly understood and deserve further exploration.

#### Acetylcholine

Progressive presynaptic cholinergic denervation is a prominent pathological manifestation of AD, accompanied by decreased choline acetyltransferase activity and acetylcholine concentrations in the brain. These abnormalities closely correlate with cognitive deficits in AD. In addition to mediating synaptic signal transmission, acetylcholine interacts with α7-nAChR to protect neurons against neurotoxicity induced by amyloid-β and tau pathology [220]. On the other hand, a growing body of evidence has shown that acetylcholine stimulation of α7-nAChR on macrophages could remarkably attenuate systemic inflammation response [172]. Further studies demonstrated that cholinergic T cells within the spleen but not cholinergic nerve cells are the source of acetylcholine that stimulates α7-nAChR on splenic macrophages to mediate the anti-inflammatory effect. Considering the observations of a low smoking prevalence among hospitalized COVID-19 patients as well as uncontrolled cytokine storm in severe COVID-19, it is postulated that dysfunction of the nicotinic cholinergic system plays a role in the pathophysiology of COVID-19 [221, 222]. Recently, the decline of acetylcholine production in the brains of AD patients is hypothesized to contribute to the elevation of COVID-19 mortality, although the influence of AD on peripheral cholinergic T cells is still unclear [13]. Therefore, the involvement of acetylcholine in the mechanistic link between AD and COVID-19 and the molecular mechanisms need to be elucidated in further studies.

#### Others

In addition to the abovementioned contributors, other potential factors underlying the mechanistic links between AD and COVID-19 are being explored. Recently, the presence of amyloid-β_1-42_ is reported to enhance the binding of the Spike protein S1 subunit of SARS-CoV-2 to ACE2, which facilitates the viral entry and exaggerates production of pro-inflammatory cytokines [223]. Consequently, increased plasma concentrations of amyloid-β in AD patients contribute to the increased morbidity and mortality of COVID-19 in AD. Moreover, data from human brain tissues have shown that the protein level of ACE2 in AD patients is upregulated compared to that in cognitively normal controls [224]. This makes AD patients more predisposed to CNS infection and adverse outcomes by the SARS-CoV-2 virus. Therefore, disruption of the ACE/ACE2 balance in AD is postulated as a contributor to the infection and severity of COVID-19 [198]. Taken together, an in-depth exploration of shared pathogenic mechanisms between AD and COVID-19 is warranted in the future, which could also in turn provide new insights into the identification of biomarkers to track disease progression and treatments of AD.

## Conclusion

There is an interaction between COVID-19 infection and neurodegenerative diseases. On the one hand, COVID-19 infection causes worsening of symptomatic severity and acceleration of neurodegeneration in PD and AD. On the other hand, neurodegenerative diseases increase the susceptibility to COVID-19 and enhances the risks of hospitalization and death after viral infection. The COVID-19 pandemic has profoundly changed the way of medical care; telemedicine services, vaccination, and specific drug therapies are promising measures for better management of PD and AD patients. Many potential molecular and cellular pathways are hypothesized to be the link between COVID-19 infection and neurodegenerative diseases. However, it is still unclear if the SARS-CoV-2 virus promotes a neurodegenerative process. Current evidence only partly but not entirely supports a relationship between SARS-CoV-2 infection and development of PD/AD. International efforts are needed to verify the relationship of SARS-CoV-2 with PD/AD.

## Data Availability

Not applicable.

## Abbreviations

^18^F-FDOPA: ^18^F-Fluorodopa
α-syn: Alpha-synuclein
ACE2: Angiotensin-converting enzyme 2
ACEI: Angiotensin converting enzyme inhibitor
AD: Alzheimer’s disease
APOE: Apolipoprotein E
ARB: Angiotensin II receptor blockers
ARIA-H: Amyloid-related imaging abnormalities-hemosiderin deposition
BBB: Blood–brain barrier
CCB: Calcium channel blocker
CI: Confidence interval
COVID-19: Coronavirus disease 2019
CSF: Cerebral spinal fluid
CVST: Cerebral venous sinus thrombosis
DBS: Deep brain stimulation
FDA: Food and Drug Administration
HR: Hazard ratio
IFN-γ: Interferon-γ
MDS: Movement disorders society
MoCA: Montreal cognitive assessment
MRI: Magnetic resonance imaging
nAChR: Nicotinic acetylcholine receptor
NLRP3: Nucleotide-binding oligomerization domain-like receptor containing pyrin domain 3
NRF2: Nuclear factor erythroid 2-related factor 2
OAS1: Oligoadenylate synthetase 1
OR: Odds ratio
PD: Parkinson’s disease
PET: Positron emission tomography
ROS: Reactive oxidative species
SARS-CoV-2: Severe acute respiratory syndrome coronavirus 2
TMPRSS2: Transmembrane serine protease 2
UPDRS: The unified Parkinson disease rating scale
VITT: Vaccine-induced immune thrombocytopenia and thrombosis
