# Correction: Links between COVID-19 and Parkinson’s disease/Alzheimer’s disease: reciprocal impacts, medical care strategies and underlying mechanisms

**DOI:** 10.1186/s40035-023-00349-x

**Published:** 2023-05-12

**Authors:** Pei Huang, Lin-Yuan Zhang, Yu-Yan Tan, Sheng-Di Chen

**Affiliations:** 1 Department of Neurology and Institute of Neurology, Ruijin Hospital, Shanghai Jiao Tong University School of Medicine, Shanghai, 200025 China; 2 Department of Neurology, Shanghai General Hospital, Shanghai, 200080 China; 3 Lab for Translational Research of Neurodegenerative Diseases, Shanghai Institute for Advanced Immunochemical Studies (SIAIS), Shanghai Tech University, Shanghai, 201210 China

**Correction to: Translational Neurodegeneration (2023) 12:5** 10.1186/s40035-023-00337-1

Following publication of this article [1], three errors were identified about the reference.

**Correction 1:**

136. Crocker TF, Brown L, Lam N, Wray F, Knapp P, Forster A. Information provision for stroke survivors and their carers. Cochrane Database Syst Rev. 2021;11:CD001919.

**Should be corrected as follows:**

136. Menni C, Valdes AM, Polidori L, Antonelli M, Penamakuri S, Nogal A, et al. Symptom prevalence, duration, and risk of hospital admission in individuals infected with SARS-CoV-2 during periods of omicron and delta variant dominance: a prospective observational study from the ZOE COVID study. Lancet. 2022;399:1618–24.

**Correction 2:**

“**Lina** and colleagues reported that 51% of patients with VITT present with cerebral venous sinus thrombosis (CVST) (95% CI 36–66%) [188].” **should be corrected to** “**Palaiodimou** and colleagues reported that 51% of patients with VITT present with cerebral venous sinus thrombosis (CVST) (95% CI 36–66%) [188].”

188. Palaiodimou L, Stefanou MI, Katsanos AH, Aguiar De Sousa D, Coutinho JM, Lagiou P, et al. Cerebral venous sinus thrombosis and thrombotic events after vector-based COVID-19 vaccines: a systematic review and meta-analysis. Neurology. 2021;97:e2136–47.

**Correction 3:**

“In human induced pluripotent stem cell (iPSC)-derived neurons and astrocytes, **Shi** and colleagues found that APOE ε4/ε4 genotype induces an increased rate of SARS-CoV-2 infection than the APOE ε3/ε3 genotype. Moreover, APOE4 astrocytes exhibited a more severe response following SARS-CoV-2 infection. This study provides the first insight into a possible APOE-mediated mechanism of COVID-19 vulnerability and severity [211].” **should be corrected to** “In human induced pluripotent stem cell (iPSC)-derived neurons and astrocytes, **Wang** and colleagues found that APOE ε4/ε4 genotype induces an increased rate of SARS-CoV-2 infection than the APOE ε3/ε3 genotype. Moreover, APOE4 astrocytes exhibited a more severe response following SARS-CoV-2 infection. This study provides the first insight into a possible APOE-mediated mechanism of COVID-19 vulnerability and severity [211].”

211. Wang C, Zhang M, Garcia G Jr, Tian E, Cui Q, Chen X, et al. ApoE-isoformdependent SARS-CoV-2 neurotropism and cellular response. Cell Stem Cell. 2021;28:331–42.e5.

The original article [1] has been corrected.
